# Catalytic Selective Oxidation of β-O-4 Bond in Phenethoxybenzene as a Lignin Model Using (TBA)_5_[PMo_10_V_2_O_40_] Nanocatalyst: Optimization of Operational Conditions

**DOI:** 10.3390/molecules28176368

**Published:** 2023-08-31

**Authors:** Juan Díaz, Luis R. Pizzio, Gina Pecchi, Cristian H. Campos, Laura Azócar, Rodrigo Briones, Romina Romero, Eduardo Troncoso, Camila Méndez-Rivas, Victoria Melín, Juan C. Murillo-Sierra, David Contreras

**Affiliations:** 1 Facultad de Ciencias Químicas, Universidad de Concepción, Concepción 4070386, Chile; juandiazs@udec.cl (J.D.); gpecchi@udec.cl (G.P.); ccampos@udec.cl (C.H.C.); rominaromero@udec.cl (R.R.); etroncoso@udec.cl (E.T.); cammendez@udec.cl (C.M.-R.); victoriamelin@udec.cl (V.M.); juanmurillo@udec.cl (J.C.M.-S.); 2 ANID—Millennium Science Initiative Program—Millennium Nuclei on Catalytic Process towards Sustainable Chemistry (CSC), Santiago 8970117, Chile; 3 Centro de Investigación y Desarrollo en Ciencias Aplicadas Dr. Jorge J. Ronco, Universidad de La Plata, La Plata B1900AJK, Argentina; lrpizzio@quimica.unlp.edu.ar; 4 Centro de Energía, Departamento de Química Ambiental, Facultad de Ciencias, Universidad Católica de la Santísima Concepción, Concepción 4090541, Chile; lazocar@ucsc.cl; 5 Centro de Investigación de Polímeros Avanzados (CIPA), Concepción 4051381, Chile; r.briones@cipachile.cl

**Keywords:** polyoxometalate nanocatalyst, Keggin-type, lignin model compound, β-O-4 bond, heterogeneous catalysis, green chemistry

## Abstract

The catalytic oxidation of phenethoxybenzene as a lignin model compound with a β-O-4 bond was conducted using the Keggin-type polyoxometalate nanocatalyst (TBA)_5_[PMo_10_V_2_O_40_]. The optimization of the process’s operational conditions was carried out using response surface methodology. The statistically significant variables in the process were determined using a fractional factorial design. Based on this selection, a central circumscribed composite experimental design was used to maximize the phenethoxybenzene conversion, varying temperature, reaction time, and catalyst load. The optimal conditions that maximized the phenethoxybenzene conversion were 137 °C, 3.5 h, and 200 mg of catalyst. In addition, under the optimized conditions, the Kraft lignin catalytic depolymerization was carried out to validate the effectiveness of the process. The depolymerization degree was assessed by gel permeation chromatography from which a significant decrease in the molar mass distribution Mw from 7.34 kDa to 1.97 kDa and a reduction in the polydispersity index PDI from 6 to 3 were observed. Furthermore, the successful cleavage of the β-O-4 bond in the Kraft lignin was verified by gas chromatography–mass spectrometry analysis of the reaction products. These results offer a sustainable alternative to efficiently converting lignin into valuable products.

## 1. Introduction

Lignocellulosic biomass is a readily available, renewable, and biodegradable resource that has attracted increasing interest with regard to producing clean energy and value-added chemicals [1]. This biomass is composed mainly of cellulose, hemicellulose, and lignin. Cellulose is a linear polymer of glucose molecules with ether linkages, whereas hemicellulose contains heteropolymers composed primarily of xylose and mannose monomers. Lignin is a complex three-dimensional polymer formed by the disordered polymerization of phenylpropane monomers with hydroxyl or methoxyl substituents [2]. Cellulose and hemicellulose are common feedstocks used in the production of paper, sugars, and biofuels [3,4]. Nevertheless, lignin, a major byproduct of these industrial activities, has not been efficiently used for chemical production, being mainly employed for bioenergy generation.

Because lignin is a complex macromolecule with a large variability of bond types, designing a specific catalyst to transform it is a complex task. Therefore, it is necessary to use model compounds to study the catalytic systems, particularly in the elucidation of lignin reaction pathways. These model compounds are especially useful, and they mainly consist of monomeric and oligomeric (mostly dimeric) phenylpropane structures [3,5,6]. In lignin, about 60–75% of the bonds between structural units correspond to ether bonds [7] and, within those, the β-O-4 ether bond, significantly weaker than the rest, corresponds to 45–62% of all types of bonds. Therefore, most studies on the mechanisms of the catalytic depolymerization of lignin focus on the cleavage of the β-O-4 bond [3]. Thus, we can establish that the lignin macromolecule is susceptible to a wide range of chemical transformations, and its fragmentation reactions can be divided into catalytic cracking, hydrolysis, reduction, and oxidation [8,9]. Recent research has established that the development of robust and selective catalytic processes specifically designed for efficient lignin conversion will be key in a lignin valorization program; this should be a central effort, such as in the petrochemical industry [10,11,12]. Among the technologies nowadays available, selective catalytic oxidation is proposed for obtaining oxidized lignin monomers such as syringaldehyde, vanillin, and vanillic acid [13]. The selective catalytic oxidation process has been studied in vapor and liquid phase systems; liquid-phase systems are more favorable since they are carried out under mild conditions of pressure and temperature in comparison with the gas-phase ones [14]. 

In liquid-phase oxidation, the choice of the oxidant determines the feasibility and efficiency of the catalytic system. According to the literature, the aerobic system (O_2_) is the ideal one and, therefore, the most widely used for lignin catalytic oxidation [15]. Molecular oxygen O_2_ has been considered the most environmentally friendly oxidant because it is cheap, easy to handle, and efficient, with water being the only by-product [16,17]. In recent years, many catalysts have been studied for this purpose; among them, polyoxometalates (POM) have attracted attention and have shown high catalytic activity in the oxidation of various target molecules [14]. Several studies have shown that the redox catalytic activity of Keggin-type polyoxometalates can be improved with Mo atom substitution by V in their primary structure [18]. This effect can be explained by considering the increases in the oxidation potential due to the substitution of Mo^6+^ by V^5+^ in the POM structure, owing to the higher reducibility of vanadium [19,20]. It has been determined that in the catalytic cycle, the substrate is firstly oxidized, and the V^5+^ of POM is reduced to V^4+^, then, the cycle is completed by the reoxidation of vanadium by O_2_ [21]. Additionally, the similar diameter of phenylpropanoid units in lignin (~1.1 nm) [22] to the POMs pore diameter is an extra feature. For instance, Liu et al. [23] successfully used Keggin-type tungstophosphoric acid (H_3_PW_12_O_40_ or 12–TPA) to oxidize lignin alkali and concluded that the unique characteristics of TPA, functioning as an acid catalyst and as a redox catalyst, favored the lignin oxidation reaction. On the other hand, its nanometric dimension allows a better interaction with the substrate and good dispersion in the reaction media. As reported in our previous work, the disubstituted V^5+^ POM showed interesting properties, such as higher crystallinity than the former precursor and thermal stability evidenced in the TGA characterization. Moreover, the V^5+^ modification produced a better dispersion of particles, generating a higher specific surface area, which is beneficial for the catalytic conversion of the substrate [24].

The main goal of this study was to carry out the catalytic oxidation of phenethoxybenzene as a dimeric model to study the selective oxidation of the β-O-4 bond in the lignin. Additionally, the optimization of the operational conditions of the process using the TBA_5_[PMo_10_V_2_O_40_] nanocatalyst was carried out. The optimization model was validated in triplicate and, after that, Kraft lignin was used as a substrate to prove the effectiveness of the process in real conditions. The results of this study offer a new strategy for developing efficient and cost-effective catalytic systems for lignin valorization.

## 2. Results and Discussion

### Statistical Analysis

The model for the screening design was significant at the 95% confidence level, and the coefficient of determination R^2^ was 0.984 (Table S1). The significance of the coefficients (β) is related to the significance of the variable (X) for the response (conversion %). The results (Table 1) showed that only the time and temperature variables (X1 and X2) had a significant effect on the conversion % (*p* ˂ 0.05), whereas the O_2_ pressure and the catalyst load did not affect the conversion. These results agree with those obtained in similar studies that used polyoxometalates in the catalytic valorization of biomass, in which time and temperature were the main factors in the process [25,26].

The slightly negative coefficients of the model are possibly due to the proximity to the zero point of the design to a maximum of the model, such that when it was increased in absolute value, the effect of the variable decreases. This observation is supported by a previous study by Reichert et al. [27] involving the aerobic catalytic oxidation of model lignocellulosic biomass substrates with H_8_[PV_X_Mo1_2-X_O_40_] type heteropolyacids, where it was found that neither the variation of O_2_ reaction pressure nor the stirring rate had a significant influence on the reaction.

The simultaneous optimization of the reaction’s time (X1), temperature (X2), and catalyst load (X3) was performed using a CCC-circumscribed central composite design, and the results are depicted in Table 2. In the previous screening study, it was determined that temperature and time were statistically significant variables. To these variables, the catalyst load (which is one of the non-significant variables) was added in this study to optimize this variable interaction with X1 and X2. Also, it is possible that the X3 variable was not significant because the maximum value was close to the central point (0) in the screening. According to the preliminary screening study, the oxidant (O_2_) pressure was set at 5 bar for optimization. The analysis of variance (ANOVA) for the second-order model is shown in Table S2. The lack of fit was found to be not significant (*p* > 0.05), indicating that the fitted model adequately represents the data. The regression was significant at a 95% confidence level, with an R2 value of 0.948.

The experimental data were fitted to a second-order polynomial equation using the polynomial model for phenethoxybenzene conversion that can be written as follows (Equation (1)):

(1) $$\mathrm{Conversion}\;\left(\%\right)=\beta_{0}+\sum_{i=1}^{k}\beta_{i}X_{i}+\sum_{i=1}^{k}\beta_{\mathrm{ii}}X_{i}^{2}+\sum_{i}\sum_{i<j=2}^{k}\beta_{\mathrm{ij}}X_{i}X_{j}+e_{i}$$

where β (0 = intercept, i = linear, ii = quadratic, and ij = interaction) and X_i_, X_j_ (i = 1, 3; j = 1, 3; and i ≠ j represents the coded independent variables) are the coefficients of the model. With the fitted quadratic polynomial equation, contour plots were developed to analyze the interaction between the terms and their effects on the phenethoxybenzene conversion performance.

Figure S1A displays the residuals versus the predicted values, which indicate a random distribution of residuals. Figure S1B shows a normal probability plot of the residuals, revealing a straight line that indicates that the residuals were normally distributed. These results demonstrate that the model accurately fits the data. Table 3 shows the estimated coefficients for the second-order model. The *p*-values obtained for the coefficients of the regression analysis indicate that the quadratic terms X1^2^, X2^2^, and X3^2^ were significant at the 95% level.

The final equation of the response model in terms of the factors (variables) can be expressed as shown in Equation (2), based on the values obtained for the statistically significant coefficients:

(2) $$\mathrm{Conversion}\;\left(\%\right)=69.47+0.06\cdot X_{1}-0.62\cdot X_{2}+0.30\cdot X_{3}-3.54\cdot X_{1}^{2}+5.86\cdot X_{2}^{2}-3.01\cdot X_{3}^{2}$$

The negative quadratic coefficients obtained are consistent with the results obtained by Shatalov et al. [28] in a system similar to ours. In their study, the authors used polyoxomolybdovanadate catalysts for the catalytic oxidation of hemicelluloses and found that the quadratic terms of temperature (−), time (+), and catalyst load (−) were significant. The presence of significant quadratic terms indicates that the response surface has a maximum, which corresponds to the optimal value of the surface. 

The negative value of the temperature coefficient can be attributed to the possibility of the over-oxidation of the substrate and/or its products. This effect was analyzed by Li et al. [29] in an oxidation reaction of lignocellulose with ionic liquids (IL) polyoxometalates ([MIMPS]_2_H_4_P_2_Mo_18_O_62_). Their work showed that since the oxidation process of these substrates is slightly exothermic, it is necessary to work at low temperatures to avoid slowing down the reaction rate and inhibiting it. The positive coefficient of the reaction time indicates that increasing the reaction time leads to higher substrate conversion (%).

The negative coefficient of the catalyst load can be explained by the presence of side reactions that generate by-products, which could limit the maximum conversion of the reaction to 75.8%, as observed in the present study. The optimum reaction conditions were determined by maximizing the conversion (%) of phenethoxybenzene using the regression model (Equation (1)), and the resulting conditions were 136.6 °C, 3.5 h, and 200 mg of nanocatalyst, which generated 75.8% of phenethoxybenzene conversion.

**Figure 1:**
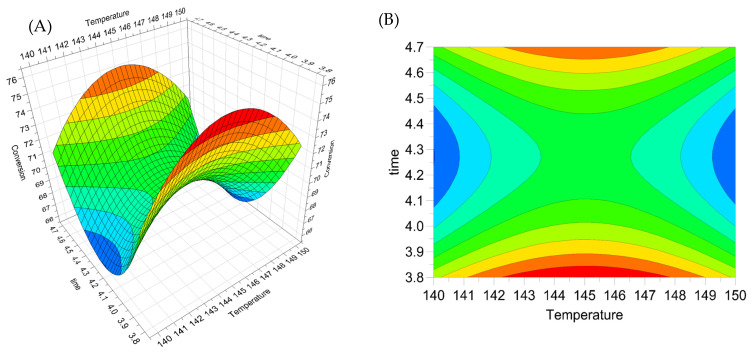
(**A**) Response surface for the catalytic conversion (%) of phenethoxybenzene as a function of time (h) and temperature (°C). (**B**) Contour plots of the conversion (%) of phenethoxybenzene predicted from the model (each contour level is equivalent to 1% of conversion). Both plots are presented at a constant catalyst load (Mcat) of 200 mg.

Based on the model, the quadratic terms of temperature (X1^2^), time (X2^2^), and catalyst load (X3^2^) were found to be the main factors affecting the conversion of phenethoxybenzene, whereas the linear terms and interactions were not significant. The response surface shown in Figure 1A and contour plots in Figure 1B were generated to illustrate the optimal combination of factors that produce the highest conversions of phenethoxybenzene.

**Figure 2:**
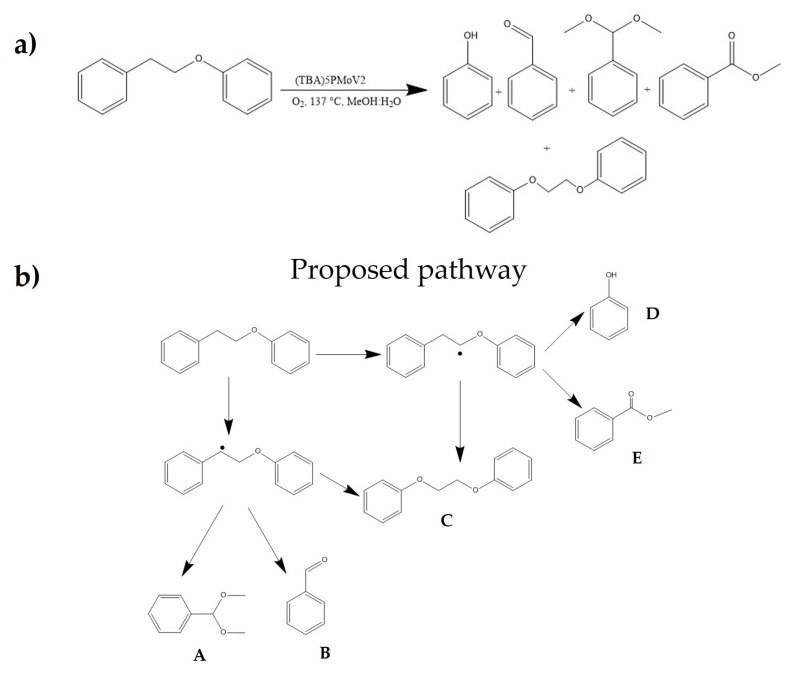
(**a**) Catalytic oxidation of phenethoxybenzene and (**b**) proposed reaction pathway.

It is noteworthy that the maximum conversion of the response surface is at the edge of the response surface (Figure 2a). A local maximum could not be found in the studied interval of time, however, according to the response surface graph, it is plausible that a high conversion could still be attained in a shorter time. To validate the mathematical model obtained, an experiment was conducted under the optimum operating conditions predicted by the response surface methodology (RSM). The conditions and results obtained from the experiment are presented in Table 4. A phenethoxybenzene conversion of 76.7% was observed under the predicted conditions by the RSM. This value is slightly higher than that predicted by the model (75.8%), but it is within the accepted tolerance range of 73.2% to 78.4%. Thus, we can conclude that the model is appropriate to describe the experimental data with a 95% confidence interval.

Under the optimized conditions, a GC–MS analysis was carried out to identify the products from the phenethoxybenzene conversion. In Figure 2, a scheme with the reaction process (a) and the proposed oxidation pathway (b) are depicted. As can be seen, all products come from the breaking at β-O-4 and α positions yielding monomeric products A, B, D, and E. Eventually, the activated units could lead to the condensation to produce C.

Table 5 shows a comparison between the proposed methodology for the catalytic oxidation of the β-O-4 bond of phenethoxybenzene with others reported in the literature for similar substrates containing the β-O-4 bond. By comparing the methodologies reported in the literature, it is observed that the optimized catalytic system proposed in this work is competitive with those previously reported. As can be seen from Table 5, a relatively high conversion percentage (up to 76.7%) was reached, and compared with similar systems, a relatively short time is needed to accomplish high conversion. On the other hand, compared to the reports with higher conversions using ionic liquids, or lasting up to 168 h, in this work, the reaction could be carried out in a shorter time with a conventional mixture of solvents, reducing technical costs. Additionally, it is a highly stable catalyst, as demonstrated in our previous study, in which, after several cycles of reuse in the benzyl alcohol oxidation, the structure and catalytic activity was maintained [24]. This suggests that our system could be an efficient and sustainable alternative for the catalytic oxidation of β-O-4 bonds in lignin model compounds.

**Figure 3:**
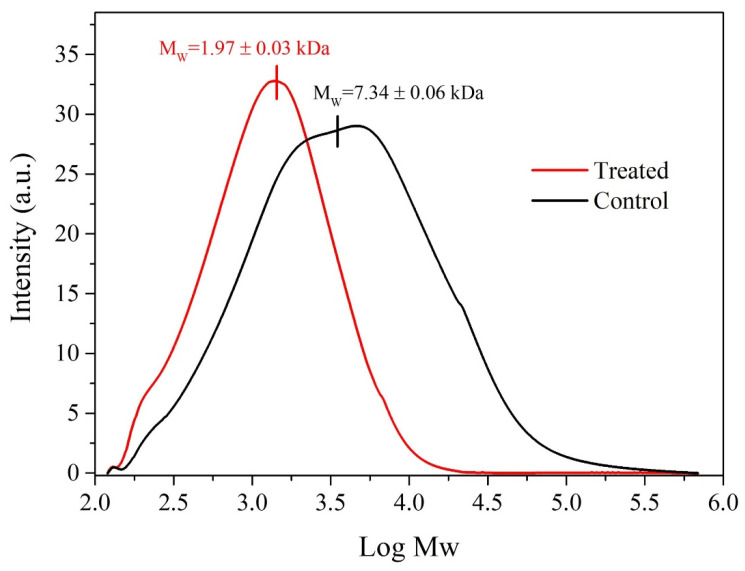
GPC chromatograms of control Kraft lignin (black) and treated Kraft lignin (red).

To assess the feasibility of the process, the catalytic selective oxidation of the β-O-4 bonds was tested in Kraft lignin depolymerization. The experiment was carried out under the validated optimal conditions, and the reduction in molecular weight and polydispersity, as well as the determination of depolymerization products, were used to evaluate the efficiency of the process. The determination of the molecular weight distribution and polydispersity index is crucial for determining the depolymerization degree of lignin. Figure 3 displays the elution profiles obtained for control and catalytically treated lignin after the derivatization treatment. As can be seen, after the catalytic process, the Kraft lignin underwent a significant transformation by reducing the weight average molecular weight (Mw) from 7.34 ± 0.06 to 1.97 ± 0.03 kDa, indicating a significant depolymerization degree. 

Additionally, it was observed that after the treatment, not only was the molar mass distribution modified, but the PDI was also greatly reduced by half from 6.00 ± 0.05 to 3.00 ± 0.30, suggesting that the depolymerization process produces selective bond breaking, obtaining a more homogenous distribution of molecular weights. These findings further confirm the effectiveness of the proposed catalytic system in the conversion of lignin.

**Figure 4:**
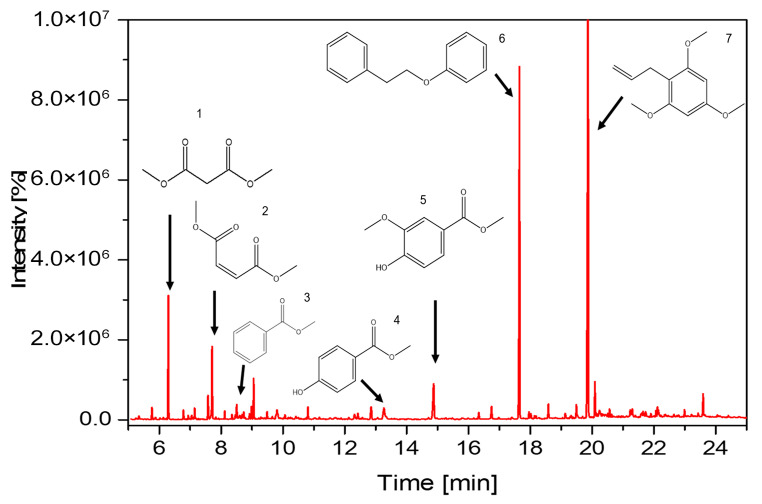
GC–MS chromatogram of products obtained from hydrothermal aerobic catalytic oxidation of lignin and some identified compounds: 1. Dimethyl malonate, 2. 2-Butenedioic acid (Z)-, dimethyl ester, 3. Benzoic acid, methyl ester, 4. p-hydroxybenzoic acid methyl ester, 5. Methyl 4-hydroxy-3-methoxybenzoate, 6. Phenethoxybenzene, and 7. 1-(2,4,6-trimethoxyphenyl)-2-propene. (Complete list in Table S3).

With the aim of acquiring more insights on the mechanism involved in the Kraft lignin depolymerization process by the catalytic system, GC–MS analysis was conducted on the reaction products. The results of the identification of the reaction products are presented in Figure 4 and Table S3. As can be seen in Figure 4, different monomers and dimers were observed as the main reaction products, with a predominance of 1-(2,4,6-trimethoxyphenyl)-2-propene and phenethoxybenzene.

The β-O-4 bonds in lignin consist of two types of bonds: C-C and C-O. These bonds connect the two benzene (aryl) rings and can be broken to generate functional groups such as aldehydes, ketones, and carboxyls in the resulting lignin fragment. Increasing the number of oxygen-rich functional groups in the lignin fragment can increase the distance between the π-π stacking of the aromatic rings, weakening the inter-unit forces that hold the lignin together and facilitating its dissociation [6]. The results of GC–MS analysis of the reaction products from the catalytic oxidation of lignin showed that the nature of the reaction products indicates cleavage of the β-O-4 bond, as evidenced by the abundant presence of oxidized fragments of veratryl alcohol (number 13, Table S3). Furthermore, the absence of vanillin and syringaldehyde among the reaction products indicates that there is a predominantly heterolytic β-O-4 bond-breaking mechanism [35]. 

In the process of breaking the β-O-4 bond, the products obtained include phenols, ethers, and aromatic esters, which were generated while maintaining the integrity of the aromatic ring in most cases. Compared to C-C bonds, C-O bonds are more prone to breakage due to the abundance of hydroxyl and ether groups in the aromatic rings and aliphatic chains of lignin, where C-O ether bonds are the most susceptible to break [36]. Additionally, studies such as that of Li et al. [37] suggest that the cleavage of C-O bonds is facilitated by catalysts with Lewis’s acid activity, which would thermodynamically favor the process. Although products obtained from the cleavage of the C-C bond, specifically the β-β bond, can be observed, such as 2-Propenoic acid, 3-(4-hydroxyphenyl)-, methyl ester (number 14, Table S3), they are present in smaller amounts. Studies of the catalytic oxidation of lignin model compounds with POMs have shown that the conversions of C-O bonds are five times higher than those of the C-C bond [37]. Aromatic ring cleavage has also been observed, generating C_6_ dicarboxylic acids (mainly muconic acid), which are unstable and are rapidly converted to C_4_ and C_2_ dicarboxylic acids, which are relatively more stable [38]. Among the dicarboxylic acids found are the esters of malonic acid (number 2, Table S3), maleic or 2-butene-dioic acid (number 3, Table S3), and succinic or 2-butanedioic acid (number 4, Table S3), which have been reported to have a high prevalence in lignin oxidation [38]. All reaction products were found in their ether or ester form rather than their form with the hydroxyl-OH functionality. 

This phenomenon was studied by Voitl et al. [39], who established that since lignin degradation with POMs generates radical intermediates, it could lead to repolymerization by condensation, inhibiting the efficiency of lignin degradation. One way to prevent these lignin–lignin condensation reactions in aqueous solvents is the inclusion of low molecular weight alcohols such as methanol. The mechanism by which methanol acts starts with the generation of dimethyl ether (DME) catalyzed by POM, then, the homolytic cleavage of the C-O bond of DME generates •OCH_3_ and •CH_3_ radicals. These radicals act as radical traps that couple with the lignin fragments before repolymerization occurs, generating the respective esters or ethers. This contradicts what was previously inferred from the analysis of the reaction products on the heterolytic mechanism. These two assertions can be reconciled if a two-stage mechanism is proposed, where the first step is the generation of the lignin fragment via heterolysis, and in the second step, the radical that will give way to the corresponding ether or ester is added. This is why POMs have been used in esterification or trans-esterification reactions [40]. The analysis of the reaction products supports the decrease in molar mass values in the catalytic depolymerization of lignin, indicating the formation of low molecular weight products and shedding light on the depolymerization reactions occurring within the reaction [36,41].

## 3. Materials and Methods

### 3.1. Materials

Phenethoxybenzene standard (97%, AmBeed, Arlington Heights, IL, USA) and all solvents (HPLC Grade, Merck, Darmstadt, Germany) were used in their commercial form without further modification. The synthesis of the catalyst TBA_5_[PMo_10_V_2_O_40_] was carried out following the reported methodology in our previous study [24]. In brief, the synthesis of the nanocatalyst TBA salt of the Keggin-type phosphomolybdodivanadate was carried out by hydrothermal method. In a typical preparation, a stoichiometric mixture of precursors, 0.01 mol H_3_PO_4_, 0.01 mol of V_2_O_5_, and 0.11 mol of MoO_3_, was dissolved in 150 mL of deionized water. subsequently, the solution was transferred to the autoclave and maintained at 80 °C with continuous stirring for 4 h. After cooling to room temperature, all the insoluble residues were removed by filtration, and the solution was heated again to 60 °C, followed by the addition of tetrabutylammonium bromide (0.012 mol). Finally, the precipitated nanoparticles were recovered by filtration and dried under vacuum until constant weight (±0.002 g). The full characterization of the catalyst can be found in our previous report [24].

### 3.2. Reaction Conditions

**Figure 5:**
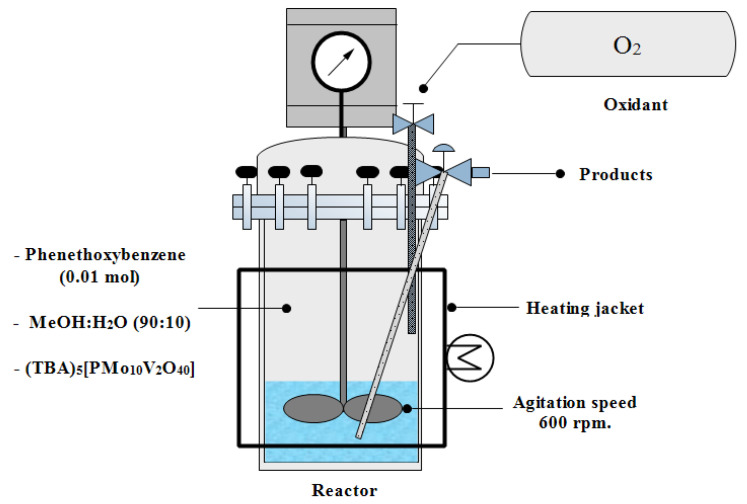
Scheme of semi-batch catalytic oxidation reactor.

The experiments were performed in an O_2_ atmosphere in a semi-batch stainless steel hydrothermal reactor with methanol: water (90:10 using *v*/*v*) as solvent (Figure 5). In a typical reaction, 1 g (0.01 mol) of phenethoxybenzene, the catalyst load according to the experimental design (100, 150, or 200 mg), and 20 mL of the solvent were added to the reactor. Subsequently, the reactor was purged three times with O_2_ (99.5%, Linde, Concepción, Chile) and pressurized with the same gas to reach the desired O_2_ concentration (2, 4.5, or 7 bar). Finally, the pressurized reactor was heated with a heating rate of 10 °C min^−1^. The selected stirring speed was 600 rpm to avoid undesired diffusive control of the reaction. 

### 3.3. Analytical Methodology

The phenethoxybenzene concentration was determined using an HPLC–DAD (Perkin Elmer, Flexar) with a mobile phase composition of H_2_O (A), Acetonitrile (B), and Methanol (C) with a flow rate of 1.0 mL min^−1^. The elution was carried out in gradient mode as follows: Step 1: 0–2 min A (50%), B (30%), and C (20%). Step 2: 2–10 min A (10%), B (30%), and C (60%). Step 3:10–15 min A (10%), B (30%), and C (60%). Step 4: 15–20 min A (50%), B (30%), and C (20%). A ZORBAX Eclipse XDB–C18 column, 4.6 × 15 mm, 3.5 µm, was used as the stationary phase, and the wavelength for detection was 260 nm. The conversion of phenethoxybenzene (X_Phenethoxybenzene_) was calculated according to Equation (3):

(3) $$X_{\mathrm{Phenethoxybenzene}\left(\%\right)}=\frac{{\left[\mathrm{Phenethoxybenzene}\right]}_{i}-{\left[\mathrm{Phenethoxybenzene}\right]}_{t}}{{\left[\mathrm{Phenethoxybenzene}\right]}_{i}}\times 100$$

where the subindices i and t refer to the initial concentration and the concentration in the time “t”, respectively. The depolymerization degree of Kraft lignin was determined by comparing the molar mass distribution of the treated sample with the untreated one. The molecular mass distribution of the samples was determined by gel permeation chromatography (GPC) using an SY-8100 UV-Vis detector (at 254 nm) with a binary LC Pump 250 (Perkin Elmer, Waltham, MA, USA) and LC Oven 101 (Perkin Elmer). Three styrogel columns 7.8 mm × 300 mm (Waters Co., Milford, MA, USA) were used as the stationary phase, and tetrahydrofuran (THF) HPLC grade was used as the mobile phase. The working flow rate was 0.5 mL·min^−1^ and the column temperature was maintained at 40 °C by a heating furnace. The data analysis was performed using the chromatography data handling system-Peak ABC 1.0 software. 

The lignin was previously derivatized (acetylation) before its injection into GPC to increase its solubility in THF [42,43]. Thus, 20 mg of untreated Kraft lignin (and 5 mL of the treated sample) were added into 2 mL of pyridine and 2 mL of acetic anhydride (1:1% *v*/*v*) with slight agitation for 20 h. Subsequently, 40 mL of miliQ water was added to the previous suspension and centrifuged at 4000 rpm for 10 min. After that, the supernatant was removed, and the solid was dried in an oven at 105 °C for 2 h. Once the sample was dried, 1 mL of THF was added, passed through a 0.22 µm filter, and injected into the GPC. Finally, the molecular weight was calculated from the calibration curve of polystyrene standards with a molecular weight range of 78–2,112,000 g mol^−1^, and the reaction products were confirmed by GC–MS using a GC model 7890A (Agilent, Santa Clara, CA, USA) coupled to a 5975C single quadrupole mass spectrometer (triple-axis detector).

### 3.4. Statistical Analysis

The optimization of the operational conditions for the catalytic aerobic oxidation of phenethoxybenzene was carried out by an experimental design based on RMS methodology. For this purpose, firstly, a fractionated factorial screening design was performed for the determination of statistically significant operating variables. A four-variable (n = 4) fractional factorial multiple linear regression model (MLR) was used, for which 11 experiments (2^4−1^ + 3) were performed. According to Table 6, the investigated variables were: temperature (T, °C), time (t, h), O_2_ pressure (PO_2_, bar), and catalyst load (Mcat, mg). Subsequently, these variables were optimized using a CCC optimization design. In both cases, the response was the phenethoxybenzene conversion (%). For the modeling of the experimental design (screening and optimization), MODDE 7.0 software (UMETRICS Inc./MKS Instruments, Umeå, Sweden) was used, and statistical validation was determined by ANOVA test at a 95% confidence level using the same software.

Optimization was carried out using a three-variable (n = 3) CCC experimental design in which 17 experiments (23 + 3 + 6) were performed. The variables and ranges studied in the process were temperature (T: 140 to 150 °C), time (t: 3.8 to 4.7 h), and mass of nanocatalyst used (Mcat: 150 to 250 mg). The variables were coded and normalized with unit values of −1 (defined as the lowest value) and +1 (defined as the highest value). The central point was coded as 0, and six star points (axial) were defined at 1.68 from the central point. Seventeen experiments were performed according to a circumscribed central composite model that included eight factorial design experiments (coded as levels −1 and +1), six star points (coded as levels −1.68 and +1.68), which are outside the range of the main domain simulating a circumference, and three central points (coded as level 0) to obtain the experimental standard deviation. The experimental design was optimized for a response variable, defined as the phenethoxybenzene conversion (%), and the predicted optimum was found using the simplex algorithm. 

The model equations included the first-order term to describe the main effects and the second-order term for the interactions. An analysis of variance (ANOVA) was used for the experimental results; non-significant effects were excluded from the model regression. Contour plots (two-dimensional response surfaces) of the model were used to define the optimal conditions for the β-O-4 bond cleavage of phenethoxybenzene. Each run was performed using phenethoxybenzene as the reaction substrate. To validate the model, an experiment was performed in triplicate under the optimal operational conditions. To assess the effectiveness of the process in real lignin, the depolymerization of a commercial Kraft type was carried out using the nanocatalyst (TBA)_5_[PMo_10_V_2_O_40_] at the optimum operational conditions obtained from the RSM. In this reaction, 200 mg of the nanocatalyst and 1 g of Kraft lignin (Sigma–Aldrich, Burlington, MA, USA) were dispersed in 20 mL of solvent, poured into the reactor, and subsequently heated to 137 °C. The O_2_ pressure was adjusted to 5 bar, and the reaction time was 3.5 h.

## 4. Conclusions

In this work, the catalytic selective oxidation of phenethoxybenzene as a lignin model was carried out under hydrothermal conditions using the (TBA)_5_[PMo_10_V_2_O_40_] nanocatalyst, and the operational conditions were optimized by RSM. The statistically significant variables, obtained during the screening design, according to the model were temperature (X1) and time (X2), with *p*-values ˂ 0.05. Based on the selected significant variables, the phenethoxybenzene was maximized by RSM, obtaining as the optimum conditions 137 °C, 3.5 h, and 200 mg of catalyst load yielding 75.8% of phenethoxybenzene conversion. In the end, the effectiveness of the process was assessed on the Kraft lignin catalytic oxidation showing a significant depolymerization degree evidenced by the reduction in molar mass distribution Mw and the PDI index data obtained from GPC. Additionally, the successful cleavage of the β-O-4 bond in the catalytic oxidation was also verified by the GC–MS analysis of the reaction products, in which the predominance of a heterolytic cleavage mechanism was proposed.

## Figures and Tables

**Table 1:** Regression analysis results of first-order polynomial model for the fractional factorial screening.

Term	Coefficient (β)	Standard Deviation	*p*-Value
Constant	80.8	1.5	˂0.05
X1	−0.0272	0.0042	˂0.05 *
X2	−2.96	0.23	˂0.05 *
X3	−0.115	0.084	0.24
X4	−0.0047	0.0046	0.36

* Significant at 95% confidence level.

**Table 2:** Circumscribed central composite (CCC) optimization design and obtained response.

Run	Variable Levels			Conversion
	X1 T [°C]	X2 t [h]	X3 Mcat [mg]	[%]
1	140	3.8	150	68.1
2	150	3.8	150	71.4
3	140	4.7	150	67.4
4	150	4.7	150	67.6
5	140	3.8	250	69.7
6	150	3.8	250	68.4
7	140	4.7	250	67.9
8	150	4.7	250	69.7
9	137	4.3	200	60.4
10	153	4.3	200	58.5
11	145	3.5	200	62.7
12	145	5.1	200	66.0
13	145	4.3	116	60.1
14	145	4.3	284	61.8
15	145	4.3	200	70.9
16	145	4.3	200	68.1
17	145	4.3	200	69.4

**Table 3:** Analysis of second-order polynomial regression model coefficients.

Term	Coefficient (β)	Standard Deviation	*p*-Value
Constant	69.47	0.93	˂0.05 *
X1	0.06	0.43	0.898
X2	−0.62	0.57	0.321
X3	0.30	0.43	0.525
X1X1	−3.54	0.52	˂0.05 *
X2X2	5.86	0.85	˂0.05 *
X3X3	−3.01	0.52	˂0.05 *
X1X2	0.00	0.57	1.000
X1X3	−0.38	0.57	0.538
X2X3	0.50	0.42	0.419

* Significant at 95% confidence level.

**Table 4:** Obtained values from the optimization with the RSM.

Independent Variables		Codified Variables	Optimal Value	Conversion [%]	
				Predicted	Experimental ± SD
Temperature	[°C]	X1	137	75.8 ± 2.6 *	76.7 ± 0.2 **
Time	[h]	X2	3.5		
Catalyst load	[mg]	X3	200		

* From 5 d.f. of residuals and two-tailed t-distribution with 95% confidence; ** n = 3.

**Table 5:** Comparison of the catalytic system studied with others reported in the literature for the oxidation of the β-O-4 bond in model compounds.

Catalyst	Solvent	Oxidant	Time (h)	T (°C)	P (MPa)	Conversion (%)	Ref.
K_5_[SiVW_11_O_40_]·12H_2_O	Buffer acetate sodium (pH 5.0)	SiVW_11_O_40_^5−^	1.0	25	-	31	[30]
V(salen)	CH_3_CN	air	24.0	80	0.1	37	[31]
(dipic)V(O)	DMSO	air	168	100	0.1	95	[32]
MTO	Acetic acid	H_2_O_2_	6.0	r.t	0.1	75	[33]
TBD	[BDMIm]Cl	-	2.0	130	-	67	[11]
ILs-CrCl_3_·6H_2_O	ILs + H_2_O	-	1.0	150	-	82	[34]
(TBA)_5_PMoV_2_	MeOH: H_2_O	O_2_	3.5	137	0.5	77	This work

**Table 6:** Variables and levels used in screening methodology.

Independent Variables		Coded Variables	Levels		
			−1	0	1
Temperature, T	[°C]	X_1_	100	150	200
Time, t	[h]	X_2_	3	4	5
Pressure O_2_, PO_2_	[bar]	X_3_	2	4.5	7
Catalyst load, Mcat	[g]	X_4_	100	150	200

## Data Availability

Almost all the data generated or analyzed during this study are included in this published article and its Supplementary Information file. Additional data are available from the corresponding author (dcontrer@udec.cl).

## Supplementary Materials

The following supporting information can be downloaded at: https://www.mdpi.com/article/10.3390/molecules28176368/s1, Table S1: Analysis of variance (ANOVA) for experimental results of fractional factorial screening design to obtain the statistically significant variables in the conversion of phenethoxybenzene; Table S2: Analysis of variance (ANOVA) for the quadratic polynomial model; Figure S1: (A) Residuals versus predicted values y, (B) Normal probability of the residuals; Table S3: Identification of catalytic Kraft lignin depolymerization products by GC–MS.
