# Supplementary material for: Catalytic Selective Oxidation of β-O-4 Bond in Phenethoxybenzene as a Lignin Model Using (TBA)5[PMo10V2O40] Nanocatalyst: Optimization of Operational Conditions
Source: Molecules. 2023 Aug 31;28(17):6368. doi: 10.3390/molecules28176368 (PMC10490183; doi:10.3390/molecules28176368)
Supplement: Supplementary file 1 [file molecules-28-06368-s001.zip › molecules-2529615-supplementary.pdf]

Supplementary Material

Catalytic Selective Oxidation of β-O-4 Bond in Phenethoxybenzene as a Lignin Model Using $(TBA)_5[PMo_{10}V_2O_{40}]$ Nanocatalyst: Optimization of Operational Conditions

Juan Díaz [1,2], Luis R. Pizzio [3], Gina Pecchi [1,2], Cristian H. Campos [1], Laura Azócar [4], Rodrigo Briones [5], Romina Romero [1], Eduardo Troncoso [1,2], Camila Méndez-Rivas [1], Victoria Melín [1], Juan C. Murillo-Sierra [1] and David Contreras [1,2,*]

[1] Facultad de Ciencias Químicas, Universidad de Concepción, Concepción 4070386, Chile
[2] ANID—Millennium Science Initiative Program—Millennium Nuclei on Catalytic Process towards Sustainable Chemistry (CSC), Santiago 8970117, Chile
[3] Centro de Investigación y Desarrollo en Ciencias Aplicadas Dr. Jorge J. Ronco, Universidad de La Plata, La Plata B1900AJK, Argentina
[4] Centro de Energía, Departamento de Química Ambiental, Facultad de Ciencias, Universidad Católica de la Santísima Concepción, Concepción 4090541, Chile
[5] Centro de Investigación de Polímeros Avanzados (CIPA), Concepción 4051381, Chile
* Correspondence: dcontrer@udec.cl

Table S1. Analysis of variance (ANOVA) for experimental results of fractional factorial screening design to obtain the statistically significant variables in the conversion of phenethoxybenzene.

Source	Sum of squares	Df. **	Mean squares	F-value	P-value
Model	55.6	4	13.91	59.67	0.001*
Residual Error	0.93	4	0.23	-	-

Lack of fit	0.68	2	0.34	2.78	0.265
Pure Error	0.25	2	0.12	-	-
Total	56.53	8	-	-	-
R^2= 0.984					
Adj. R^2= 0.967					

* Significant at 95% confidence level; ** Degrees of freedom

Table S2. Analysis of variance (ANOVA) for the quadratic polynomial model.

Source	Sum of squares	Df. **	Mean squares	F-value	P-value
Model	236.1	9	26.2	10.16	0.010*
Residual Error	12.9	5	2.58	-	-
Lack of fit	8.98	3	2.99	1.524	0.420
Pure Error	3.93	2	1.96	-	-
Total	249.0	14	-	-	-
R^2= 0.948					
Adj. R^2= 0.855					

* Significant at 95% confidence level; ** Degrees of freedom.

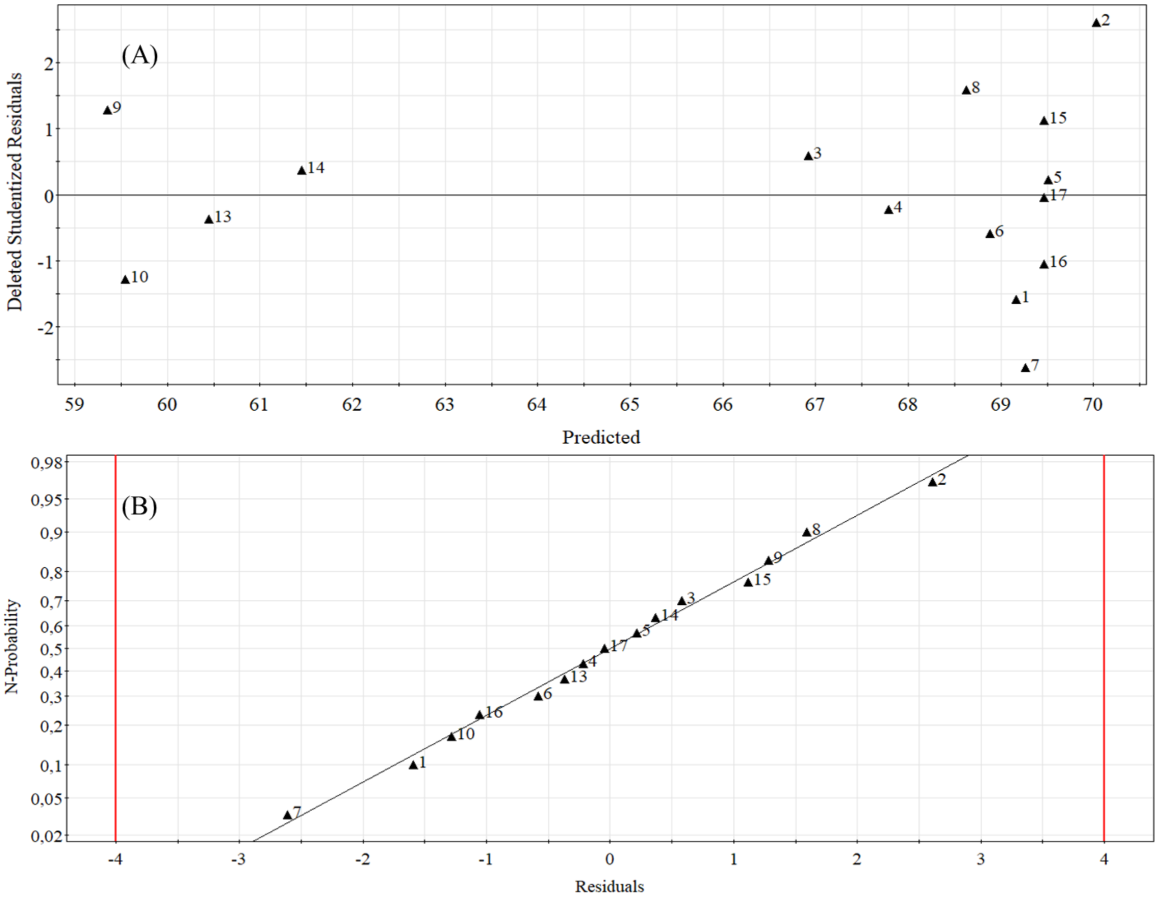

Figure S1. (A) Residuals *versus* predicted values y (B) Normal probability of the residuals.

Table S3. Identification of catalytic Kraft lignin depolymerization products by GC-MS.

Peak number	Compound name	Retention time [min]	Structure
1	1,1,3-Trimethoxypropane	5.8	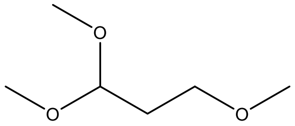
2	Dimethyl malonate	6.3	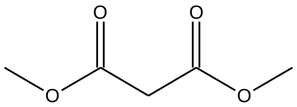
3	2-Butenedioic acid (Z)-, dimethyl ester	7.6	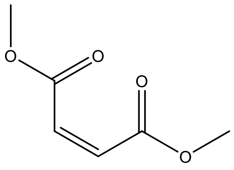
4	2-Butanedioic acid dimethyl ester	7.7	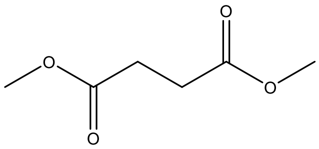
5	Benzoic acid, methyl ester	8.5	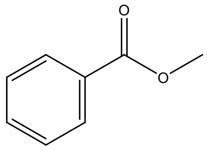
6	2-Isopropoxypropanol	8.9	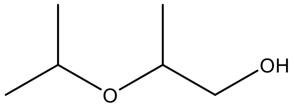
7	Butanedioic acid, methoxy-, dimethyl ester	9.1	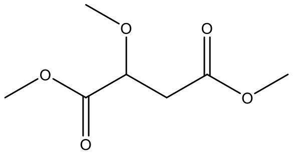
8	Tributylamine	9.8	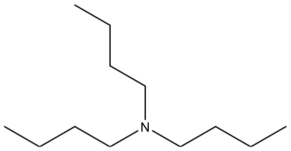

9	Trimethyl ethane-1,1,2-tricarboxylate	10.8	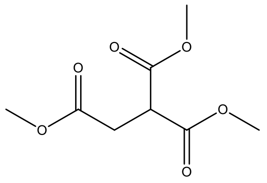
10	(1E)-1-propene-1,2,3-tricarboxylic acid 1,2,3-trimethyl ester	12.9	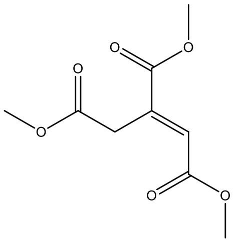
11	*p*-hydroxybenzoic acid methyl ester	13.3	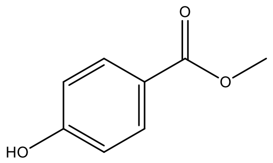
12	Methyl 4-hydroxy-3-methoxybenzoate	14.9	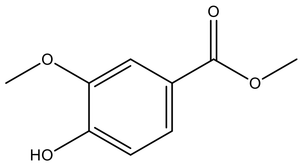
13	Methyl 3,4-dimethoxybenzoate	16.3	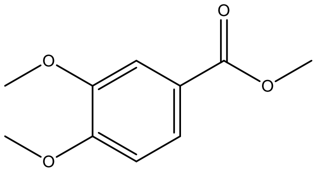
14	2-Propenoic acid, 3-(4-hydroxyphenyl)-, methyl ester	16.7	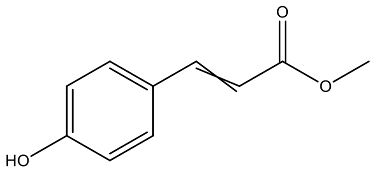
15	Phenethoxybenzene	17.7	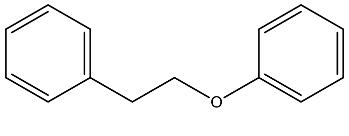
16	Methyl 3-formyl-2,4-dihydroxy-6-methylbenzoate	18.6	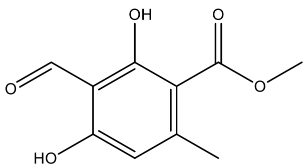

17	Trimethyl 1,2,4-benzenetricarboxylate	19.5	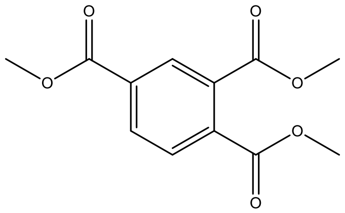
18	1-(2,4,6-trimethoxyphenyl)-2-propene	19.9	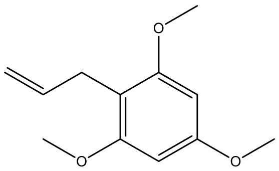
19	Dimethyl 5-aminobenzene-1,3-dicarboxylate	20.1	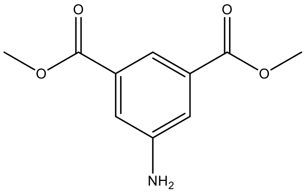
20	4H-Benzo[def]naphtho[2,3-b]carbazole	23.6	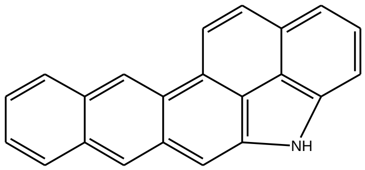
